# Maculopathies made simple

**Published:** 2025-01-31

**Authors:** David Yorston

**Affiliations:** 1 Consultant Ophthalmologist, Tennent Institute of Ophthalmology, Gartnavel Hospital, Glasgow, Scotland.

**Macular diseases are on the increase worldwide.**

**Figure:**
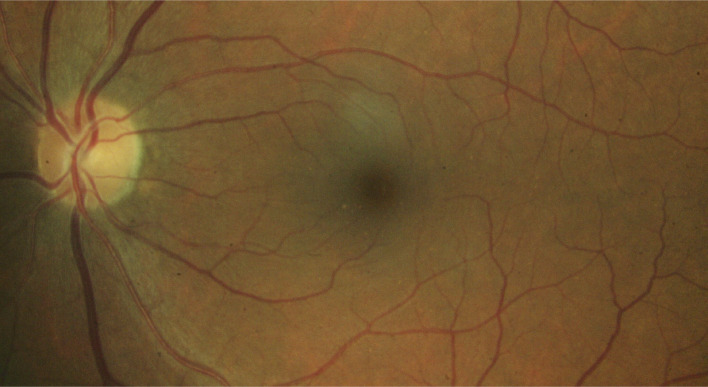
The normal macula.

© DAVID YORSTON CC BY-NC-SA 4.0

The macula is the region of the retina with the highest density of photoreceptors and is responsible for our central vision. Whenever we read print, recognise a face, or examine a patient's eye, we are using our macula. Damage to the macula makes everyday tasks, such as reading and recognising faces, much more difficult.

In this issue of the *Community Eye Health Journal*, we are focusing on the most common and treatable conditions affecting the macula:

- Diabetic macular oedema
- Age-related macular disease
- Surgical maculopathies: epiretinal membrane and macular hole
- Myopic maculopathy.

## Why this issue, why now?

Macular diseases are on the increase worldwide. Diabetes is increasing more rapidly in low- and middle-income countries compared to high-income countries^1^ along with its complications, including diabetic macular oedema. Age-related macular degeneration (AMD) – as its name suggests – tends to affect older people. With more people living to an older age, the number of people with AMD is projected to grow from 195 million in 2020 to over 288 million by 2040.^2^ It was the 3rd most common cause of moderate to severe visual impairment worldwide in 2015, after cataract and refractive error, and the 4th most common cause of blindness globally.^3^ Myopia is also increasing rapidly worldwide and, with it, complications such as myopic maculopathy.^4^

Macular diseases – particularly exudative macular degeneration and diabetic maculopathy – respond well to intravitreal injections of anti-VEGF drugs, such as bevacizumab. Anti-VEGF drugs are still expensive, but the recent World Health Organization Package of Eye Care Interventions (PECI) specifically includes recommendations for the use of anti-VEGF drugs, which should make it easier to advocate for their inclusion in national eye health programmes and insurance coverage.

Surgical maculopathies, such as macular hole and epiretinal membrane, can be treated using vitrectomy, and the outcomes are good. Patients with myopic maculopathy can benefit from anti-VEGF injections for macular new vessels; traction maculopathy can be improved using a scleral buckle (see page 18).

## When to suspect a macular condition

The macula is responsible for the detailed vision that is assessed when we measure visual acuity. If the macula has a significant disorder, the visual acuity in that eye will be reduced. If the visual acuity is 6/12 or better, the patient may have a mild problem – early atrophic AMD, or a small epiretinal membrane – but it certainly doesn't need treatment or referral.

If the vision is less than 6/12, the patient may have macular disease, but cataract and refractive error are more common, so look for those first. The patient may also have glaucoma or another condition affecting the optic nerve. If there is no evidence of such conditions, then you can suspect a macular problem (Figure 1).

One very helpful pointer to macular disease is distortion, or metamorphopsia. If a patient complains that straight lines look bent, or distorted, it is almost certain that they have a macular problem. Not all macular conditions cause distortion – it is uncommon in atrophic AMD – so the absence of symptoms of distortion does not exclude macular disease. Patients rarely volunteer symptoms of distortion, so, if you suspect a macular disorder, ask the patient to look at a straight line, such as a doorway or window frame, and to report any distortion.

## What macular diseases should I be able to recognise?

**Figure 3:**
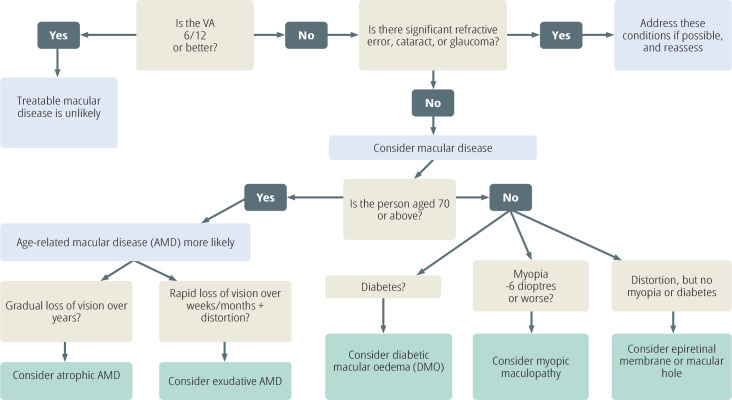
Flow diagram for identifying macular conditions.

The flow diagram in Figure 3 will get you started. This is just a pointer to what is the most likely diagnosis. People with diabetes can get AMD, and macular holes. People with high myopia can have diabetic maculopathy or AMD. However, if someone has no history of diabetes, and normal blood sugar, it is extremely unlikely that they have diabetic maculopathy. If someone has a refractive error of -1.5, it is extremely unlikely that they have myopic maculopathy. If there is no distortion, they don't have a macular hole. People over 60 can get AMD, but it is much more common in people over the age of 70.

Once you have narrowed down the likely diagnoses, you need to examine the macula. You must dilate the pupil in order to do so. If an eye has severe visual impairment (6/60 or worse) the abnormality of the macula is usually obvious. If there is only mild visual impairment, the signs can be more difficult to detect, but it is almost always possible to identify an abnormal macula (see page 4). These patients should be referred, ideally to a centre able to provide OCT examination (see page 15).

Most patients with macular conditions will still have some visual impairment. It is therefore important to support them to make the best use of their vision, by providing advice and referring them to low vision and rehabilitation support, where available. Patients may benefit from training in eccentric viewing (using peripheral vision). The Macular Society offers free online training for patients: www.macularsociety.org/support/daily-life/skills-seeing/evtraining/
